# An analysis of the São Silvestre race between 2007–2021: An increase in participation but a decrease in performance

**DOI:** 10.1016/j.smhs.2023.03.007

**Published:** 2023-03-31

**Authors:** Mabliny Thuany, Douglas Vieira, Elias Villiger, Thayse Natacha Gomes, Katja Weiss, Pantelis T. Nikolaidis, Caio Victor Sousa, Volker Scheer, Beat Knechtle

**Affiliations:** a CIFI2D, Faculty of Sports, University of Porto, 4200-450, Porto, Portugal; b Post-Graduation Program of Physical Education, Federal University of Sergipe, São Cristóvão-SE, Brazil; c Institute of Primary Care, University of Zurich, 8091, Zurich, Switzerland; d Department of Physical Education and Sport Sciences, University of Limerick, Limerick, Ireland; e School of Health and Caring Sciences, University of West Attica, Athens, 12243, Athens, Greece; f Bouve College of Health Sciences, Northeastern University, Boston, MA, 02115, USA; g Ultra Sports Science Foundation, 109 Boulevard de l'Europe, 69310, Pierre-Benite, France; h Medbase St. Gallen Am Vadianplatz, St. Gallen, Switzerland

**Keywords:** Endurance, Performance, Sex differences

## Abstract

This study aimed to investigate the trends of finishers in the São Silvestre race in Brazil, taking into account sex, age, and performance levels. A total of 31 ​775 runners (women, *n* ​= ​13 ​847; men, *n* ​= ​17 ​928), aged (45.2 ​± ​16.8) years, finishers in the São Silvestre race between 2007 and 2021, were considered in the present analysis. Data (event year, date of birth, sex, and race times) were downloaded from the official race website. The man-Whitney *U* test, Spearman correlation, and robust regression model were computed. Participation increased over time for both sexes. Regarding age groups, “31–40 years” (women) and “> 60 years” (men) were those with the highest number of finishers. We found a decrease in performance across the years (*β* ​= ​2.45; *p* ​< ​0.005), as well as significant differences in race times for both sexes (*U* ​= ​42.844; *p* ​< ​0.001), with men presenting better performances than women. Over time, it was observed an increase in the performance gap between the sexes, but in general, the performance decreased (*β* ​= ​1.76; *p* ​< ​0.001). Stakeholders should consider improving the strategies to improve women and young people's participation in running events.

## Abbreviations

*CI*: Confidence interval
*Δ*: Difference
*SD*: Standard deviation
y: Year

## 1 Introduction

The number of running events increased worldwide in the last few years. Data from the USA showed that participation in 5 ​km running events between 2000 and 2016 increased. Previous data showed that in 2019 approximately 459 029 people completed a marathon.^1^ Meanwhile, in Brazil the number of runners and running events has increased since the 1970s.^2^^,^^3^ Dr. Cooper played a relevant role in this increase through the use of his physical training methods for the Brazilian soccer team during the training for the world cup in 1970.^4^ Nowadays, running is one of the main physical activities performed by the Brazilian population.^5^^,^^6^ A national report indicated that ∼4.1% of Brazilians practice running.^5^ In addition, the country characteristics, which included specific environmental characteristics (i.e., parks, beaches), natural characteristics (i.e., annual temperature, humidity, precipitation, light), and tourism growth contributed to the increase of this phenomenon. A previous study showed that many Brazilian runners traveled to the Brazilian Southeast region (e.g., São Paulo, Rio de Janeiro, and Minas Gerais) to participate in running events.^7^^,^^8^ More than half of the Brazilian population lives in this region, which is the region with the highest PIB in the country and also concentrates the highest number of sports events, including running.^8^ The state of São Paulo, the most populated Brazilian state, has experienced an increase in the number of runners and events over time.^9^

The São Silvestre race is the Brazilian oldest and most prestigious road-based running event. Since 1924, the race has been held on the last day of the year (December 31st) and features both high-performance athletes and recreational runners from different countries. The race has been held in the São Paulo state since 1924. In the first event, the race had 60 athletes registered, of which 48 finished the event. As the event grew over the years, the organization limited the number of participants. In 1948, the organization started to hold preliminaries throughout the country, where each state could send their athletes best positioned in the ranking to compete in São Silvestre, but the state of São Paulo could have 250 athletes attending the event. In 1975, women competed for the first time in São Silvestre, and the first female winner was the German Christa Valensieck. In 1991, a change in the course was performed, from 12.6 ​km to 15 ​km, and the race was included in the street competition calendar of the International Association of Athletics Federations (now World Athletics). The increase in the number of participants reached its peak in 2014 with 30 ​000 runners. In 2020, for the first time since its begging, the race did not happen due to the COVID-19 pandemic.

Information about participation, performance trends, and sex differences was previously shown for different race distances such as half-marathon and marathon,^10^, ^11^, ^12^, ^13^ sex,^14^ age groups,^15^ performance levels,^16^^,^^17^ and country of origin.^18^, ^19^, ^20^ The studies showed an increase in runners’ participation, especially for women, younger and older runners.^21^, ^22^, ^23^ However, this increase in participation was related to a decrease in the performance^11^^,^^24^ for both sexes, which reduced the performance gap between the sexes.^25^^,^^26^ However, despite the tradition of this event in Brazil, no information is available regarding the participation, performance, and trend in sex differences in the São Silvestre race. This study aims to: (a) analyze the participation and performance trends considering the sex and age groups of finishers of the São Silvestre race, and (b) verify the performance gap between men and women. We hypothesized an increase in participation among both younger and older athletes and a decrease in performance over the years. Based on previous findings that showed overall stability of the sex gap in long-distance events,^25^ we also hypothesized that men would present a better performance compared to women, but the performance gap would be decreasing over the years.

## 2 Methods

### 2.1 Ethical approval

This study was approved by the Institutional Review Board of Kanton St. Gallen, Switzerland, with a waiver of the requirement for informed consent of the participants as the study involved the analysis of publicly available data (EKSG 01-06-2010).

### 2.2 Study design and data source

The study used a cross-sectional design. All data were collected in January 2022 from the official results section. Despite the race being first held in 1924, available results comprised the period between 2007 and 2021 and included information up to the first 100 finalists in each age group in each year. Downloaded information included event year, date of birth, sex, and race time. The runners’ age was computed using the date of birth and the date of the competition. Age categories were considered (18–20 years [y]; 21–30 ​y; 31–40 ​y; 41–50 ​y; 51–60 ​y; >60 ​y), and the running pace was estimated by the ratio “time conclusion/running distance (15 ​km)”. For the analysis, the pace was used as “s/km”.

### 2.3 Statistical analysis

Descriptive information was presented as mean ​± ​standard deviation (*SD*) and frequency (%). Data normality was tested through the Kolmogorov-Smirnov test and visual Q-Q-plot. Considering the non-parametric distribution, the Mann-Whitney *U* test was used to verify sex differences in race time. Sex differences were computed in delta values over time (2007–2021). The Spearman correlation coefficient was computed for both sexes to explore a potential relationship between age, year, race time, and running pace. A robust linear regression model was estimated considering the running pace as the outcome variable and sex (female/male), age groups (18–20, 21–30, 31–40, 41–50, 51–60, > 60), and event year (2007–2021) as independent variables. All statistical analyses were performed in SPSS 22 and STATA (V.14), considering a confidence interval (*CI*) of 95%, and a significance level of (*p* ​< ​0.05).

## 3 Results

Data comprises 31 ​775 official finishers of the São Silvestre race (women: 13 ​847; men: 17 ​928) during 2007–2021 with a mean age of (45.2 ​± ​16.8) y (women: [43.2 ​± ​14.3] y; men: [46.8 ​± ​18.3] y). The average pace was 338 ​s/km (women: [384 ​± ​71] s/km; men: [301 ​± ​85] s/km). The mean race time was ([1 ​h 29 ​min 55 ​s] ​± ​[26 ​min 31 ​s]). Significant differences were shown for race time for both sexes (*U* ​= ​42.844; *p* ​< ​0.001), with men presenting better performances ([1 ​h 18 ​min 53 ​s] ​± ​[24 ​min 45 ​s]) compared to women ([1 ​h 44 ​min 12 ​s] ​± ​[21 ​min 24 ​s]).

### 3.1 Participation over time

Table 1 presents the race finishers considering both sex and age categories. Most of the finalists were aged between 31 and 40 ​y and >60 ​y for women and men, respectively. The lowest number of finishers was found among the youngest athletes (18–20 ​y) (women: 498; men: 1 542). Considering both sexes together, an increase in the number of finishers was found over time, with the lowest value in 2007 (women: 819; men: 1 212; total athletes: 2 031 athletes) and the highest value in 2019 (women: 1 101; men: 1 343; total athletes: 2 444 athletes).

**Table 1:** São Silvestre race finalists by sex and age categories.

		2007	2008	2009	2010	2011	2012	2013	2014	2015	2016	2017	2018	2019	2021	Total *per* age-group
Woman	*18–20 ​y*	31	26	39	32	33	29	43	44	33	39	40	40	33	27	489
	*21–30 ​y*	209	213	198	204	201	202	206	204	209	210	201	203	204	173	2 837
	*31–40 ​y*	204	206	206	197	224	218	203	206	206	198	206	201	205	214	2 894
	*41–50 ​y*	197	206	200	209	199	197	210	199	204	201	202	209	205	197	2 835
	*51–60 ​y*	178	180	202	202	197	199	188	205	200	206	205	195	201	207	2 765
	*> 60 ​y*	0	71	79	110	107	143	182	159	166	172	176	179	253	230	2 027
Men	*18–20 ​y*	114	119	110	113	115	114	115	112	118	108	112	114	118	60	1 542
	*21–30 ​y*	209	196	209	205	201	202	205	202	199	206	209	210	198	211	2 862
	*31–40 ​y*	196	204	204	204	211	212	205	204	210	203	204	198	203	200	2 858
	*41–50 ​y*	209	203	205	206	203	198	205	213	194	201	201	201	207	198	2 844
	*51–60 ​y*	204	203	202	209	197	200	193	191	208	203	197	208	199	203	2 817
	*> 60 ​y*	280	311	318	311	325	363	379	373	360	358	403	401	418	405	5 005
*Total per year*		2 031	2 138	2 172	2 202	2 213	2 277	2 334	2 312	2 307	2 305	2 356	2 359	2 444	2 325	

### 3.2 Performance trends

**Fig. 1:**
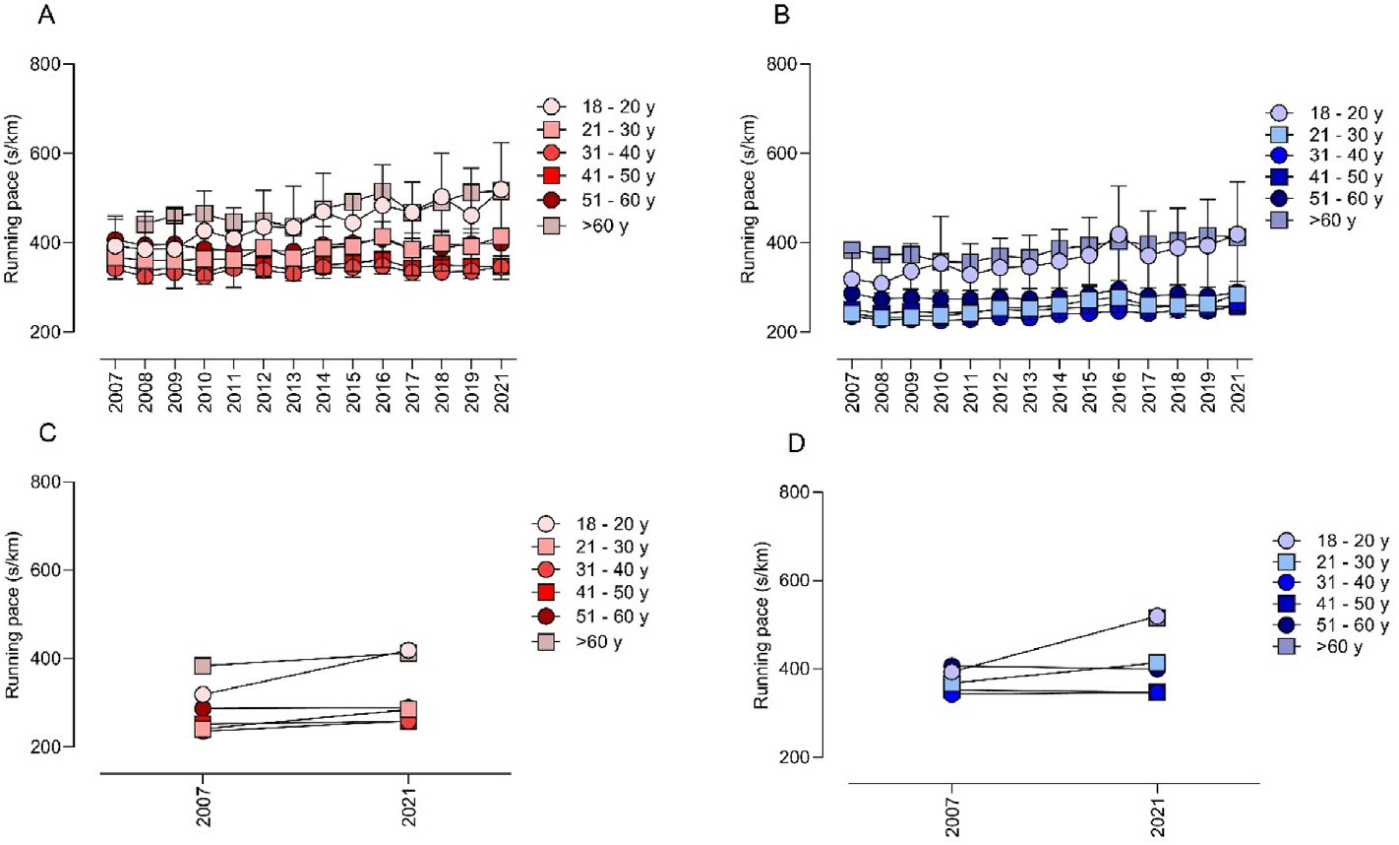
Running pace for each age group in the São Silvestre race during 2007–2021 (A) Woman; (B) Men; (C) Woman; (D) Men.

Performance over time is presented in Fig. 1 for both sexes and all age groups. When the mean running pace was considered, visual information indicated that performance decreased over time for both sexes and all age groups (Fig. 1A and Fig. 1B). Considering the first (2007) and the last (2021) years of the analysis, men aged 41–60 presented stability in performance, while performance decreased in the other age groups for both sexes. For women, younger runners (18–20 ​y) showed the highest running pace differences between 2007 and 2021 (Fig. 1, panel C).

**Fig. 2:**
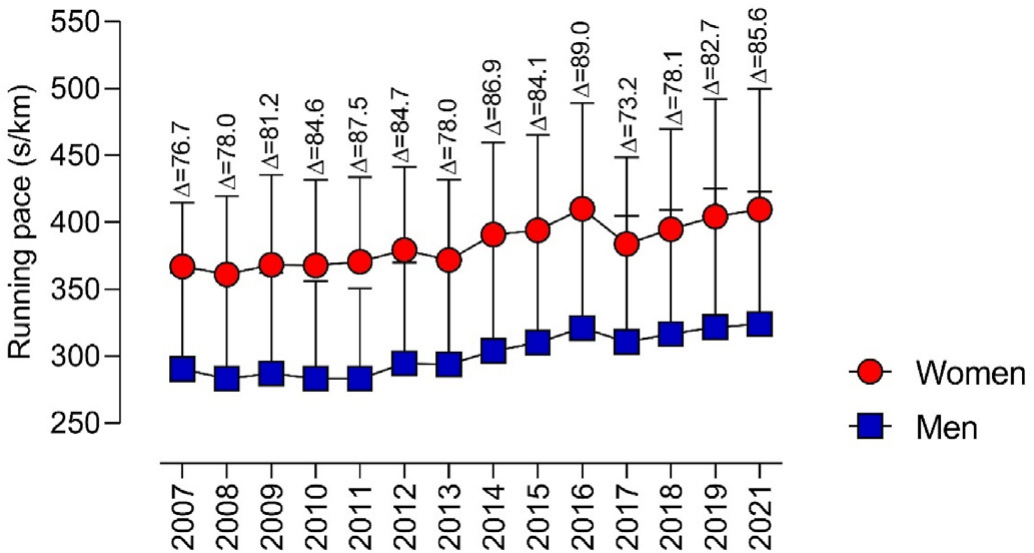
Sex differences in running performance over time.

Sex differences over time are presented in Fig. 2, based on mean values for running pace. Over time, performance differences presented a variation, with men presenting the best values compared to women. The lowest and highest performance differences were shown in 2017 (Δ ​= ​73 ​s/km) and 2016 (Δ ​= ​76 ​s/km), respectively. The last two years showed an increase in sex differences, with women presenting a worse performance than men.

**Fig. 3:**
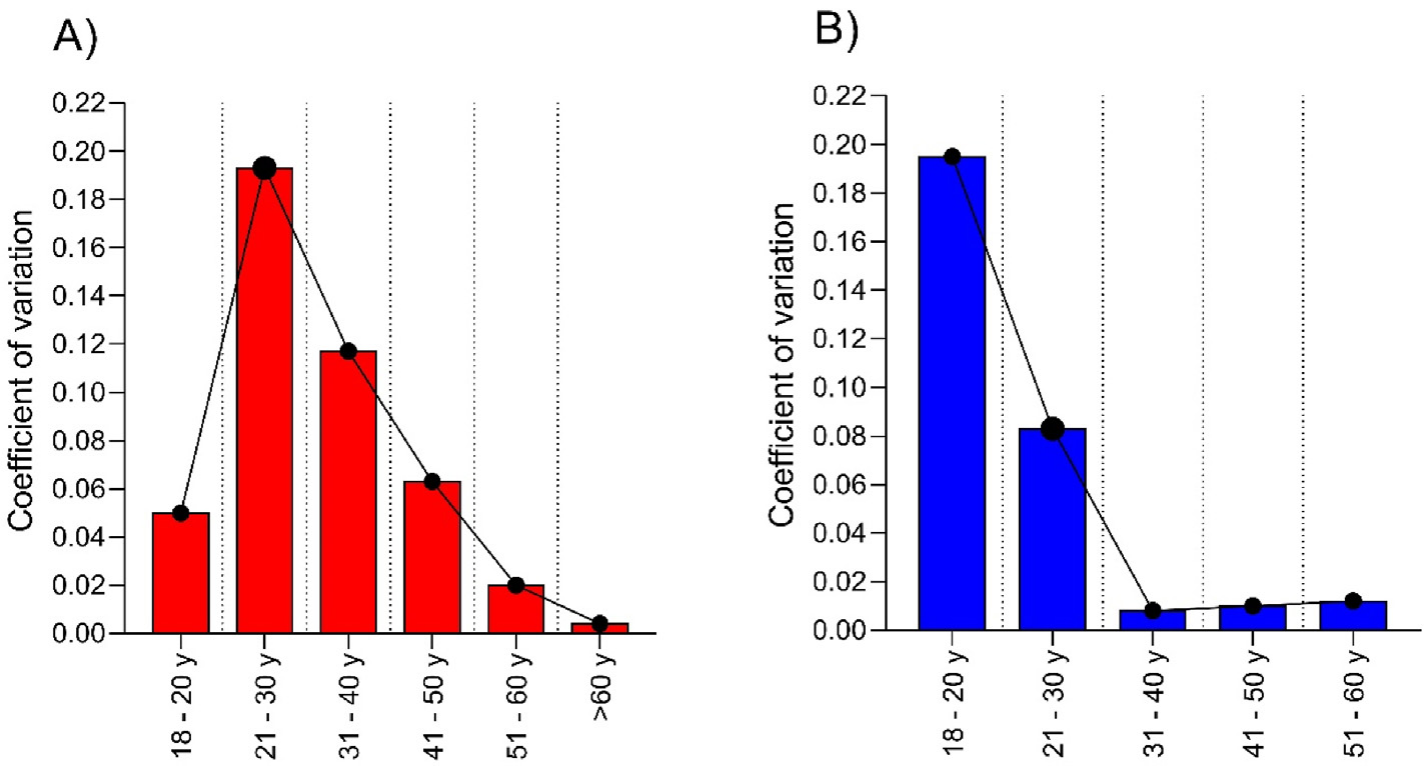
Coefficient of variation for running pace in the first (2007) and the last year (2021) for age categories in both sexes A) Women; B) Men.

Fig. 3 presents the results for the coefficient of variation for both sexes and all age groups. The highest performance heterogeneity was verified for women aged 21–30 ​y and men 18–20 ​y. The oldest women (60 ​y) and men aged 31–40 ​y presented the lowest heterogeneity.

Table 2 presents the Spearman correlation results for the relationship between year, age, race time, and running pace. For both sexes, a significant and positive association between the calendar year and race time was shown (women: *ρ* ​= ​0.037; 95%*CI* ​= ​0.021–0.054; men: *ρ* ​= ​0.166; 95%*CI* ​= ​0.153–0.181). That is, performance decreased across the years for both sexes. Similar results were shown for the relationship between age and race time for both sexes. An increase in age was positively and significantly associated with race time and running pace. Results of robust regression models showed that men presented a better performance compared to women (*β* ​= ​−94.90; *p* ​< ​0.001) in ∼94 ​s/km. An increase in age was negatively associated with performance. Over time, the runner's performance decreased (*β* ​= ​1.76; *p* ​< ​0.001).

**Table 2:** Spearman correlation results in the relationship between year and age with race time and running pace.

				Women				Men			
				Year	Age	Race time	Running pace	Year	Age	Race time	Running pace
Year	Spearman correlation			1.000	**0.104∗∗**	**0.037∗∗**	**0.185∗∗**	1.000	**0.055∗∗**	**0.166∗∗**	**0.192∗∗**
	*p*-value				**< 0.001**	**< 0.001**	**< 0.001**		**< 0.001**	**< 0.001**	**< 0.001**
	Sample size			13 847	13 847	13 847	13 847	17 928	17 928	17 928	17 928
	Bootstrap	Bias		0.000	0.000	0.000	0.000	0.000	0.000	0.001	0.001
		Standard error		0.000	0.008	0.008	0.008	0.000	0.007	0.007	0.007
		95%*CI*	Lower	1.000	0.088	0.021	0.168	1.000	0.041	0.153	0.179
			Upper	1.000	0.120	0.054	0.201	1.000	0.069	0.181	0.208
Age	Spearman correlation			**0.104∗∗**	1.000	**0.284∗∗**	**0.323∗∗**	**0.055∗∗**	1.000	**0.512∗∗**	**0.522∗∗**
	*p*-value			**< 0.001**		**< 0.001**	**< 0.001**	**< 0.001**		**< 0.001**	**< 0.001**
	Sample size			13 847	13 847	13 847	13 847	17 928	17 928	17 928	17 928
	Bootstrap	Bias		0.000	0.000	0.000	0.000	0.000	0.000	0.000	0.000
		Standard error		0.008	0.000	0.010	0.010	0.007	0.000	0.008	0.008
		95%*CI*	Lower	0.088	1.000	0.264	0.302	0.041	1.000	0.497	0.506
			Upper	0.120	1.000	0.302	0.342	0.069	1.000	0.527	0.536
Race time	Spearman correlation			**0.037∗∗**	**0.284∗∗**	1.000	**0.912∗∗**	**0.166∗∗**	**0.512∗∗**	1.000	**0.989∗∗**
	*p*-value			**< 0.001**	**< 0.001**		**< 0.001**	**< 0.001**	**< 0.001**		**< 0.001**
	Sample size			13 847	13 847	13 847	13 847	17 928	17 928	17 928	17 928
	Bootstrap	Bias		0.000	0.000	0.000	0.000	0.001	0.000	0.000	0.000
		Standard error		0.008	0.010	0.000	0.002	0.007	0.008	0.000	0.001
		95%*CI*	Lower	0.021	0.264	1.000	0.907	0.153	0.497	1.000	0.988
			Upper	0.054	0.302	1.000	0.916	0.181	0.527	1.000	0.990
Running pace	Spearman correlation			**0.185∗∗**	**0.323∗∗**	**0.912∗∗**	1.000	**0.192∗∗**	**0.522∗∗**	**0.989∗∗**	1.000
	*p*-value			**< 0.001**	**< 0.001**	**< 0.001**		**< 0.001**	**< 0.001**	**< 0.001**	
	Sample size			13 847	13 847	13 847	13 847	17 928	17 928	17 928	17 928
	Bootstrap	Bias		0.000	0.000	0.000	0.000	0.001	0.000	0.000	0.000
		Standard error		0.008	0.010	0.002	0.000	0.007	0.008	0.001	0.000
		95%*CI*	Lower	0.168	0.302	0.907	1.000	0.179	0.506	0.988	1.000
			Upper	0.201	0.342	0.916	1.000	0.208	0.536	0.990	1.000

Bold: significant value; ∗∗*p* ​< ​0.001.

## 4 Discussion

The purpose of this study was to verify the participation and performance trends considering the sex and age groups of finishers in the São Silvestre race and to examine performance differences between the sexes. The main findings showed (Ⅰ) an increase in the number of finishers over time; (Ⅱ) higher participation and faster performance for men compared to women; (Ⅲ) an increase in the performance gap between sexes over time; (Ⅳ) athletes aged 41–60 ​y presented stability in performance over time, while the youngest (21–30 ​y and 18–20 ​y for women and men, respectively) presented the highest performance variability; and (Ⅴ) a negative trend in performance over the years.

### 4.1 Participation and sex differences

The increase in participation confirms our hypothesis. An increase in the number of runners in road-based running races of different distances was presented in previous studies for different distances.^27^, ^28^, ^29^, ^30^, ^31^ Running events have been considered one of the main sports events around the world^32^ and one of the main physical activities,^33^ as well as an important tool for the World Health Organization Global Action Plan on Physical Activity 2018–2030.^34^ The use of running events as an important strategy to increase physical activity levels, are related with the benefits for social, health, and quality of life, psychological aspects,^35^ and socioeconomic factors since running is a more accessible practice compared to other activities.^32^^,^^36^

Sex differences were shown for both participation and running performance. Although the sex difference in participation has decreased over the years, the number of men participating is still higher than women, as observed in recent studies.^7^ Considering the Brazilian population, a previous study showed a higher prevalence of running practice among women.^6^ The difference in running/sports participation was previously shown and was associated with traditional gender roles (e.g., beliefs about responsibilities and behavioral expectations). Women face several barriers to participating in sports events, such as the lack of time due to the double work journey, security perception, and fear of crime.^37^

Our hypothesis that men perform better than women has been confirmed. Regarding the performance differences, the present study confirms existing scientific literature,^38^^,^^39^ showing better performance for men in all distance events.^12^ Sex differences are explained by biomechanical, physiological, morphological, motivational, and thermoregulatory differences between sexes.^40^^,^^41^ For instance, men have higher maximal oxygen uptake, while in contrast, women have greater non-sagittal hip and knee joint motion, proportional area of type I fibres, and the ability to use fatty acids and preserve carbohydrates during prolonged exercise.^40^

Besides these differences, previous studies showed that for elite athletes and ultra-endurance events, women could reduce the gap with men.^12^^,^^25^^,^^42^ However, the present study could not confirm existing findings. The gap increased over the last years, where the highest performance differences in the last years, compared to the first, can be related to race distance or motivational factors. Male Brazilian runners are twice as likely as women to engage in running due to performance aims,^43^ while most of the women report the health, aesthetic, social, and quality of life as the main factors associated with their running practice.^44^^,^^45^ These motivational differences are related to differences in training commitment and running performance between both sexes.^45^ In addition, the São Silvestre race is one of the oldest and most prestigious running events around the country, where part of the finishers participate for socialization, tourism, or leisure purposes, and not for performance improvement.

### 4.2 Variability and performance trend

We also observed that older men (41–60 ​y) presented the highest performance stability over time than their younger counterparts. Previous studies showed a higher performance variability among younger male runners (< 30 ​y) of both sexes.^13^^,^^46^ The training background and familiarity with the competition route can be related to the lower performance variation for older athletes.^46^ Previous studies have shown that the oldest runners were motivated by health, while younger athletes were motivated by the competition,^15^^,^^18^^,^^47^ which can influence pacing strategies and performance variation.

Decreasing in performance across calendar years might be partially attributed to the increased participation rates. Since more runners participated in this race, it was assumed to become more crowded, involving more recreational runners. The observation of combined increased participation and decreased performance was in agreement with studies on marathon races such as the “Berlin Marathon”^48^ and the “New York City Marathon”.^27^ Moreover, environmental factors can be related to performance variability and decreased performance over time. In the last years, the highest temperatures were shown during the race day. The association between temperature and runners' performance was previously shown,^10^^,^^49^, ^50^, ^51^ indicating that an increase in temperature tends to slow down slower runners.^51^^,^^52^ However, considering methodological differences between studies, especially the running event distance – São Silvestre race is an event that covers 15 ​km – future studies should consider the environmental characteristics (weather, precipitation, pollution) that can impair runners' performance.

This study is not free of limitations. For the present study, we considered only information available in official results, which does not cover the results of all participants, as well as data from the years before 2007 and data about elite athletes. In addition, secondary data is limited regarding individual factors associated with the performance, such as body mass, body mass index, and training experience (i.e., volume/week, intensity, methods used). Another point is that we do not have control over multiple participations over time. The São Silvestre is the oldest and most prestigious event in Brazil, and this is the first study to verify participation and performance trends. Practical implications include using this information to guide the next events and increase the participation of the sub-represented groups.

## 5 Conclusion

Participation in the São Silvestre race has increased over the last years, while performance has decreased over time. Most of the finishers were aged 31–40 ​y for women and older than 60 ​y for men. The youngest athletes showed the lowest participation and the highest performance variability over time. The performance gap increased over time. Future studies need to address the aspect of motivation for São Silvestre participation in both sexes, age groups, and performance levels.

## Submission statement

Our work submitted has not been published previously, is not under consideration for publication elsewhere, its publication is approved by all authors and tacitly or explicitly by the responsible authorities where the work was carried out, and, if accepted, it will not be published elsewhere including electronically in the same form, in English or in any other language, without the written consent of the copyright holder.

## Ethical approval statement

This study was approved by the Institutional Review Board of Kanton St. Gallen, Switzerland, with a waiver of the requirement for informed consent of the participants as the study involved the analysis of publicly available data (EKSG 01-06-2010).

## Authors’ contributions

MT conceptualized this study, conducted the literature search, perform the statistical analysis and wrote the original draft preparation. BK and TNG conceptualized this study, reviewed and edited this paper. EV organized the datasets. DV, KW, PTN, CVS, and VS reviewed and edited this paper. All authors have read and approved the final version of the manuscript and agree with the order of presentation of the authors.

## Conflict of interest

Beat Knechtle is an editorial board member for Sports Medicine and Health Science and was not involved in the editorial review or the decision to publish this article. All authors declare that there are no competing interests.
